# A New Function of the S100-A4 Protein (Mts1): Mts1 Stimulates the Activation of Cytotoxic Lymphocytes via the TREM-1 Receptor

**DOI:** 10.3390/ijms27146359

**Published:** 2026-07-17

**Authors:** Daria M. Zhelezova, Elena A. Romanova, Anna V. Tvorogova, Rustam H. Ziganshin, Denis V. Yashin, Lidia P. Sashchenko

**Affiliations:** 1 Institute of Gene Biology, Russian Academy of Sciences, Moscow 119334, Russia; yrkina121@gmail.com (D.M.Z.); elrom4@rambler.ru (E.A.R.); annatvor@mail.ru (A.V.T.); sashchenko@genebiology.ru (L.P.S.); 2 Shemyakin & Ovchinnikov Institute of Bioorganic Chemistry, Russian Academy of Sciences, ul. Miklukho-Maklaya, 16/10, Moscow 117997, Russia; ziganshin@mail.ru

**Keywords:** TREM-1, Mts1, peptides, cytotoxic lymphocytes, tumor cells, apoptosis, necroptosis

## Abstract

The search for new regulators of the immune response is an important task of modern immunology. In this work, we have found that Mts1 binds with high specificity to the innate immunity receptor TREM-1 and forms a stable complex with it. The same complex was found on the cell surface of macrophages. The appearance of soluble sTREM-1 in a conditioned medium after Mts1 interaction is considered the starting point of receptor activation. PCR analysis indicates activation of proinflammatory genes IL6, IL1β, and TNF after Mts1 administration. Based on these results, Mts1 can be considered a novel TREM-1 ligand. Using limited trypsinolisis, we have identified the epitopes of Mts1 responsible for TREM-1 activation. Similar to the full-length protein Mts1, the 17aa М7 peptide (^41^ELPSFLGKRTDEAAFQK^57^) of the Mts1 protein activates the TREM-1 receptor. Incubation of human lymphocytes with the Mts1 protein of its M7 peptide results in the appearance of cytotoxic subpopulations of NK cells and T lymphocytes, able to lyse HLA-deficient cancer cells via apoptosis or necroptosis. Activated lymphocytes induce apoptosis and necroptosis in HLA-negative tumor cells. The new regulatory peptide may be potentially used for the regulation of inflammatory processes and activation of antitumor immunity.

## 1. Introduction

Inflammatory processes play an important role in the immune response [1]. On the one hand, the induction of inflammatory receptors is a necessary step in the development of an immune response to various pathogens. On the other hand, chronic inflammation also plays an important role in pathogenesis [2,3]. Therefore, elucidating the mechanisms of interaction of inflammatory receptors with their ligands is an important task in modern immunology.

A widely studied inflammatory receptor is the TREM-1 innate immunity receptor (triggering receptor expressed on myeloid cells) [4,5]. This receptor is involved in the induction of inflammation and inflammation-related pathological processes [6,7]. TREM-1 is an enhancer of the TLR-dependent immune response. In synergy with TLR4, it is responsible for the overexpression of inflammatory cytokines (“cytokine storm”), leading to the uncontrolled development of inflammation and adverse consequences for the body [8]. Activation of TREM-1 begins with dimerization of the receptor and then develops in two ways. In the first case, the activation of the cascade of tyrosine protein kinases leads to the activation of the transcription factor NFkB, followed by the expression of genes for proinflammatory processes [9,10]. In the second case, activation of TREM-1 leads to the appearance of cytotoxic T-lymphocytes, which kill tumor cells that have escaped immune control [11]. Dimerization of the receptor is accompanied by dissociation of the soluble exodomain sTREM-1 into a conditioned medium [12,13].

A wide range of TREM-1 ligands has been described. These include actin [14], RNA-binding protein [15], glycoproteins on the cell surface of Ebola and Marburg viruses [16], and the innate immunity protein PGLYRP1 (Tag7) [17]. In our laboratory, it was shown that the ligands of TREM-1 are also the cellular protein HMGB1 [18,19], the main protein of heat shock Hsp70 [20].

Mts1 (S100A4, metastasin-1) has diverse functional activity aimed at both enhancing tumor progression and developing immune protection [21,22]. The S100A4 protein is known as an enhancer of tumor progression [23,24]. We have shown that it is involved in the binding of cytotoxic T-lymphocytes to Hsp70 on the surface of HLA-negative tumor cells. In combination with Hsp70, Mts1 activates chemotaxis of cytotoxic lymphocytes [25]. In complex with the Tag7 epitope, peptide 17.1, it participates in the killing of TNFR1-positive tumor cells [26]. Our preliminary data allow us to consider it a TREM-1 ligand.

One of the promising approaches to the study of the mechanisms of interaction of ligands with the receptor is the identification of functionally active epitopes of ligands interacting with the receptor [27]. Such shortened peptide fragments can have both activating and inhibitory activities and can be used to understand the detailed mechanism of intracellular signal induction upon ligand–receptor contact, as well as in immunotherapy [28,29,30]. The advantage of using these peptides is that the body’s own peptides carry a lower risk of developing allergic and autoimmune reactions. Epitopes of many TREM-1 ligands that activate its functional activity have been identified. Peptides included in these epitopes have been synthesized and are involved in the regulation of inflammatory processes [31,32,33].

In this work, the interaction of the Mts1 protein and its epitope with the TREM-1 receptor was studied. The main tasks were: (1) to study the interaction of the Mts1 protein and its functional peptide fragment with TREM-1 in solution and on the cell surface; (2) to identify the expression of proinflammatory cytokine genes during the interaction of Mts1 or its epitope with TREM-1; (3) to characterize the TREM-1-dependent activation of cytotoxic lymphocytes under the action of Mts1 or its epitope.

## 2. Results

### 2.1. Mts1 Interacts with TREM-1 and Activates This Receptor

Our preliminary results suggested that Mts1, interacting with TREM-1, activates cytotoxic lymphocytes. Here, we investigated the correspondence of the Mts1 structure to the active center of TREM-1. The interaction of these two proteins in solution was studied using microscale thermophoresis.

**Figure 1:**
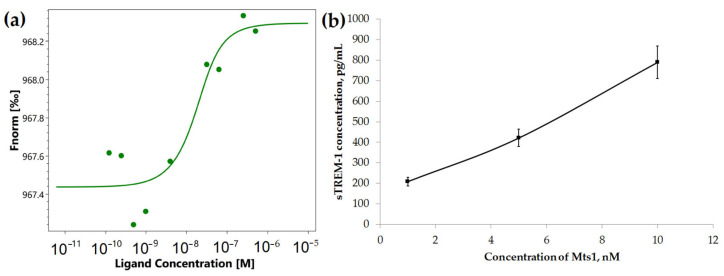
(**a**) Microscale thermophoresis data on the interaction of labeled with the Alexa Fluor™ 633 protein labeling kit sTREM-1 with Mts1. The experiment was carried out in three repetitions, showing the most common data. (**b**) ELISA analysis of RAW264.7 cells for sTREM-1 release depending on the concentration of added Mts1.

Shown in Figure 1a, the clear dependence of the thermophoretic signal on the concentration of Mts1 indicates the binding of these proteins and the formation of a stable protein complex. The calculated dissociation constant of this complex was 8.88 ± 0.12 nmol, which suggests a high affinity for the active sites of these proteins and the stability of the formed complex. Having established the possibility of forming a stable complex between Mts1 and TREM-1 in solution, it was necessary to establish such a possibility when such a complex interacts with the TREM-1 receptor on the cell membrane.

**Figure 2:**
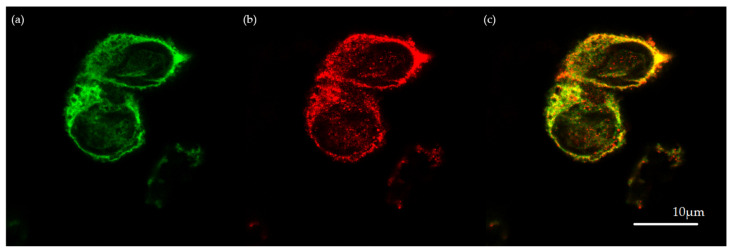
(**a**) Staining with antibodies to TREM-1 of RAW264.7 cells; (**b**) staining with antibodies to Mts1 of RAW264.7 cells; (**c**) image overlay.

Further, the interaction of Mts1 with cells of the RAW264.7 mouse macrophage line was studied using confocal microscopy (Figure 2). These cells carry the TREM-1 receptor on the cell surface and are often used in the study of inflammatory processes. The results shown in Figure 2a indicate a high expression of this receptor on the cell membrane. Statistical analysis has shown that almost all RAW264.7 cells contain TREM-1 on the cell surface. It can also be seen that Mts1 binds strongly to the cell membrane (Figure 2b). The colocalization shown in Figure 2c shows that Mts1 mainly binds to TREM-1 on the cell membrane. The number of such cells in the population is 83%. Similar results were obtained on human U937 cells (See Supplemental Figure S1). Thus, it can be assumed that Mts1 forms a complex with TREM-1 on the cell membrane. Next, we investigated how the binding of Mts1 to the TREM-1 receptor affects the activation of this receptor.

As mentioned above, activation of TREM-1 begins with dimerization of the receptor monomers, followed by dissociation of the receptor exodomain from the cell surface. The activation of TREM-1 is judged by the appearance of sTREM-1 (soluble TREM-1) in the cellular supernatant [34]. To detect the activation of TREM-1 by interaction with Mts1, an enzyme immunoassay was used to determine sTREM-1 in the supernatant (Figure 1b). It can be seen that the amount of sTREM-1 increases with increasing concentration of Mts1. Therefore, the dissociation of the TREM-1 receptor’s exodomain is dependent on the concentration of Mts1, and Mts1 can be considered a ligand of TREM-1.

Next, we investigated how Mts1-dependent activation affects the functions of this receptor: the expression of proinflammatory cytokine genes and the activation of cytotoxic lymphocytes.

### 2.2. Mts1 Induces TREM-1-Dependent Expression of Inflammatory Cytokine Genes

The expression of inflammatory cytokine genes is considered one of the main functions of macrophages in regulating the immune response [35].

**Figure 3:**
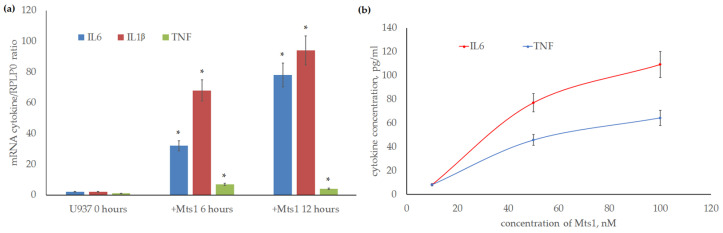
(**a**) rtPCR analysis of cytokines IL-1β, IL-6, and TNF in U937 in an isolated via magnetic separation subpopulation of monocytes after addition of Mts1 protein (n = 3 for each groups). (*p*-value: * <0.05). (**b**) ELISA analysis of secreted IL6 and TNF proteins in the conditioned medium of U937 cells after 24 h of incubation with various concentrations of Mts1 protein.

Given the limited number of macrophages in the PBMC (peripheral blood mononuclear cells) of a healthy donor, human protomonocyte line U937 cells were studied to study cytokine activation. For stimulation, these cells were incubated with PMA (phorbol esters) for 24 h. Stimulated cells were incubated with Mts1, and the levels of TNF, IL-6, and IL-1β mRNAs were determined after 6 and 12 h (Figure 3a). It can be seen that the mRNA level of all three cytokines increases significantly after 12 h of incubation, 10 times for TNF, 20 times for IL-6, and 40 times for IL-1β. To test the actual level of secreted cytokines in the cellular supernatant, ELISA tests for IL6 and TNF were used (Figure 3b). It can be seen that the level of secreted IL-6 and TNF cytokines increases after 24 h of incubation with Mts1 protein, which was dose-dependent. Consequently, Mts1 activates the ability of TREM-1 to express inflammatory cytokine genes.

### 2.3. Mts1 Induces TREM-1-Dependent Cytotoxicity of Lymphocytes

**Figure 4:**
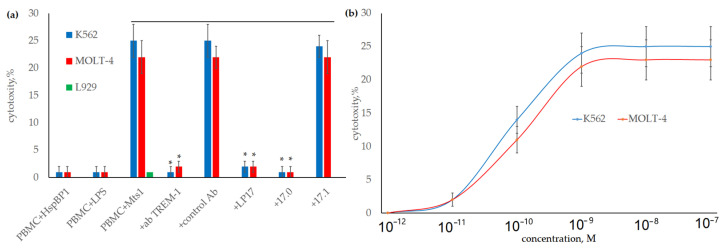
(**a**) The cytotoxic activity of PBMC activated by Mts1 (1 nM) after 20 h incubation with K562, MOLT-4 and L929 cells in the presence of an antibody to TREM-1, control antibody (both 1:100, 20 h), or the blocking peptides LP17, 17.0 of control 17.1 peptide (all 10 nM). HspBP1 protein (10 nM) or LPS from *E. coli* (10 µg/mL) failed to activate cytotoxic PBMC; (*p*-value: * <0.05). (**b**) the dependence of the cytotoxic activity of PBMC on the concentration of the added protein Mts1, measured on K562 and MOLT-4 cells.

As mentioned above, TREM-1 activates both the expression of inflammatory cytokine genes and cytotoxic lymphocytes. Next, we investigated the cytotoxic activity of lymphocytes stimulated by the interaction of Mts1 via TREM-1. For this purpose, PBMC were incubated with Mts1, and their ability to lyse HLA-negative tumor cells was determined after 6 days. The results shown in Figure 4a indicate that activated PBMC fractions have cytotoxic activity inhibited by antibodies to TREM-1, as well as by TREM-1 inhibitors—peptides LP17 and 17.0. It is obvious that Mts1 induces TREM-1-dependent activity of cytotoxic lymphocytes. The results are shown in Figure 4b demonstrate that the cytotoxic activity of lymphocytes increases linearly with increasing concentration of Mts1. The maximum cytotoxicity was achieved at a concentration of 1 nmol. The target cells for these lymphocytes were erythro- and T-lymphoma cells, K562 and Molt-4 cell lines, respectively, which do not carry HLA antigens on the cell surface. Control cells of the L-929 line are resistant to the action of Mts1-stimulated lymphocytes (Figure 4a).

### 2.4. Mts1 Activates Three Subpopulations of Cytotoxic Lymphocytes: NK Cells, CD8+ and CD4+ T Lymphocytes, and They Induce Programmed Cell Death Processes in Tumor Cells

**Figure 5:**
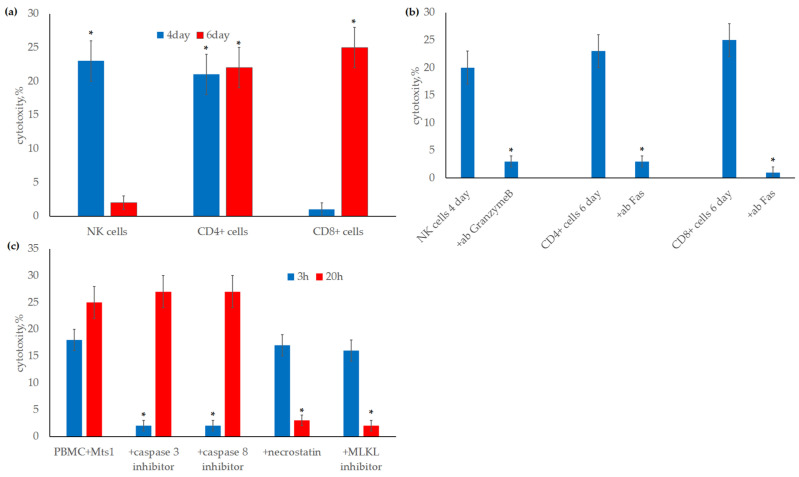
(**a**) The cytotoxic activity of purified PBMC activated by Mts1 subpopulations of NK cells, CD4+, and CD8+ T lymphocytes for 4 or 6 days, long after 20 h incubation with K562. (**b**) The cytotoxic activity of purified from PBMC activated by Mts1 subpopulations of NK cells on day 4, CD4+ and CD8+ T lymphocytes on day 6 in the presence of antibodies to GranzymeB and Fas (1:100, 20 h). (**c**) Cytotoxic activity of PBMC activated by Mts1 protein alone and in the presence of caspase 3 and 8 inhibitors, RIP1-kinase inhibitor (necrostatin1), and MLKL kinase inhibitor. (*p*-value: * <0.05).

The cytotoxic effect of Mts1-activated lymphocyte subpopulations was further characterized. It was previously shown that changes in lymphocyte cytotoxicity depending on the incubation time with the ligand are not linear, but are characterized by two maxima on the 4th and 6th days. With the use of the magnetic separation method, after 4 and 6 days of incubation, subpopulations of NK cells, as well as CD8+ and CD4+ T lymphocytes, were isolated from the total population of PBMC, and their cytotoxic activity was determined. Cytotoxicity was detected after 20 h incubation of enriched lymphocyte subpopulations with HLA-negative K562 tumor cells. Figure 5a shows the results of cytotoxicity of NK cells, CD8+, and CD4+ T-lymphocytes on days 4 and 6. It can be seen that on the 4th day, NK cells and CD4+ T lymphocytes are activated, and CD8+ T lymphocytes are inactive. On the 6th day, the cytotoxicity of NK cells disappears, but the cytotoxic activity of CD8+ T lymphocytes appears, and CD4+ T lymphocytes remain active (For flow cytometry see Supplemental Figure S2).

It has previously been shown that various mechanisms of recognition and induction of cell death are activated in NK cells and T lymphocytes. It can be seen that NK cells kill HLA-negative tumor cells by secreting granzymes [36] (Figure 5b). CD8+ and CD4+ T lymphocytes kill target cells by FasL-Fas interaction.

It is known that the Fas receptor induces two alternative cell death processes in the cell, apoptosis and necroptosis, which are activated at different time intervals of incubation with target cells [37]. Therefore, cytotoxic activity was determined after 3 or 20 h incubation of CD8+ and CD4+ T lymphocytes with tumor cells (Figure 5c). It can be seen that after 3 h, both CD8+ T lymphocytes and CD4+ T lymphocytes induce caspase-dependent apoptosis in cells, and after 20 h RIP-1 kinase and MLKL are activated in cells, which is typical for necroptotic processes. Additionally, anti-pMLKL antibodies were used to test MLKL activation in the target cells via Western blot (See Supplemental Figure S3).

### 2.5. The 17-Mer Mts1 Epitope Interacts with TREM-1 and Induces Receptor Activation

**Figure 6:**
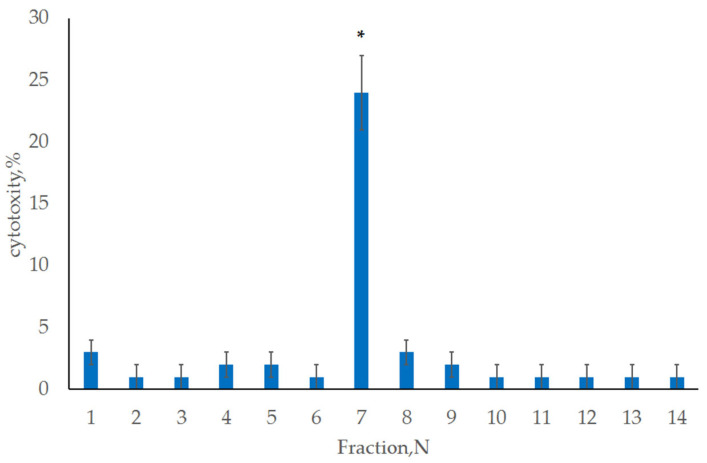
Peptide fractions of Mts1 after limited trypsinolysis were separated on Superdex Peptide column and then were added to PBMC for 6 days, and on day 6, activated lymphocytes were transferred to K562 tumor cells for 20 h to test their cytotoxic activity. (*p*-value: * <0.05).

Thus, Mts1 interacts with the TREM-1 receptor and enhances its functional activity: expression of pro-inflammatory cytokine genes and activation of cytotoxic lymphocytes. Next, it was necessary to identify the Mts1 epitope responsible for this interaction with the receptor and its subsequent activation. For this purpose, limited trypsinolysis was used, and the resulting Mts1 peptides were separated on a Superdex Peptides column. To analyze the activation ability of peptides, peptides of each fraction were incubated with PBMS and cytotoxic activity of lymphocytes was detected. The results are shown in Figure 6.

It can be seen that only the peptide of fraction No. 7 had the ability to activate cytotoxic lymphocytes. The amino acid sequence of this peptide was identified using MALDI (Matrix Assisted Laser Desorption/Ionization) analysis, and the M7 peptide (^41^ELPSFLGKRTDEAAFQK^57^) was synthesized.

**Figure 7:**
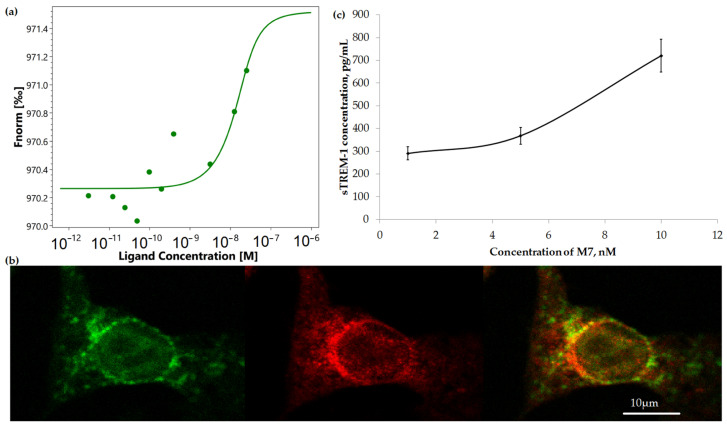
(**a**) Microscale thermophoresis data on the interaction of labeled with the Alexa Fluor™ 633 protein labeling kit sTREM-1 with M7. The experiment was carried out in three repetitions, showing the most common data. (**b**) Green staining with antibodies to TREM-1 of RAW264.7 cells; red staining with antibodies to Mts1 of RAW264.7 cells; yellow-image overlay. (**c**) ELISA analysis of RAW264.7 cells for the isolation of sTREM-1 depending on the concentration of the added M7 peptide of the Mts1 protein. 10 µm.

The ability of the 17-membered M7 peptide to bind to TREM-1 in solution and on the cell surface, as well as to initiate the dissociation of TREM-1 from the cell surface, was studied. Microscale thermophoresis has shown that the M7 peptide binds to TREM-1 with high affinity. The dissociation constant Kd is 5.32 ± 0.09 nmol (Figure 7a) and is comparable to the Kd of the interaction of Mts1 with TREM-1, these results indicate the specificity of the interaction of the peptide with the receptor and the stability of the formed complex.

The M7 peptide exhibits the same binding specificity when interacting with TREM-1 on the cell surface. The results of studies using confocal microscopy and statistical analysis indicate a high colocalization of the peptide with the receptor (78% of cells) (Figure 7b).

As mentioned above, the interaction of TREM-1 with ligands leads to dimerization of the receptor and dissociation of TREM-1 from the cell surface. It is possible to see the release of sTREM-1 into the conditioned PBMC medium after their incubation with the M7 peptide (Figure 7c).

### 2.6. The M7 Peptide Activates the Expression of Pro-Inflammatory Cytokine Genes and Cytotoxic Lymphocytes

**Figure 8:**
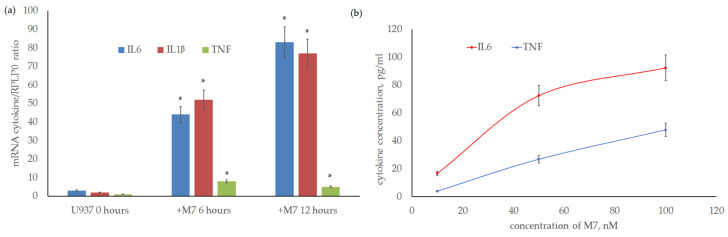
(**a**) rtPCR analysis of cytokines IL-1β, IL-6, and TNF in U937 in isolated via magnetic separation subpopulation of monocytes after addition of M7 peptide of Mts1 protein. (n = 3 for each groups). (*p*-value: * <0.05). (**b**) ELISA analysis of secreted IL6 and TNF proteins in the conditioned medium of U937 cells after 24 h of incubation with various concentrations of M7 peptide.

Next, we investigated how the interaction of the M7 peptide with TREM-1 affects the functional activity of this receptor. Figure 8a shows changes in the amount of mRNAs of inflammatory cytokines TNF, IL-6, and IL-1β depending on the incubation time of protomonocytes U937 with peptide M7. It can be seen that the amount of mRNA of each cytokine increases after 6 and 12 h of incubation of cells with the peptide. Figure 8b shows the amount of secreted IL6 and TNF cytokines, tested by the ELISA method. It can be seen that the amount of IL6 and TNF proteins increases in the cultured medium after 24 h of incubation of cells with the peptide, and is proportional to the amount of added M7 peptide.

**Figure 9:**
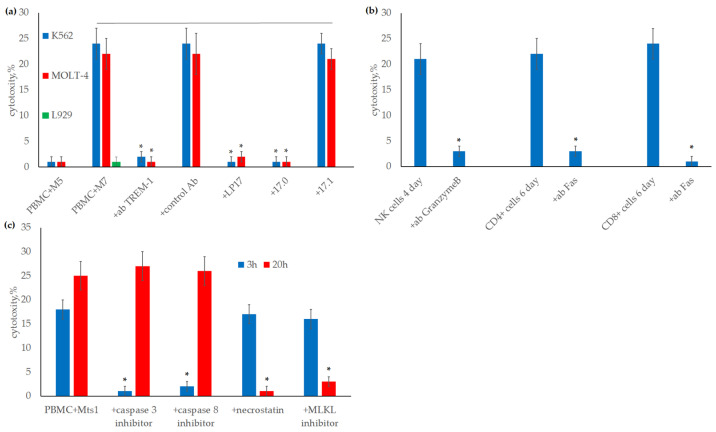
(**a**) Cytotoxic activity of PBMC activated by M7 peptide after 20 h incubation with K562, MOLT-4, and L929 cells in the presence of antibodies to TREM-1 (1:100, 20 h) or blocking peptides LP17 and 17.0 (10 nm). (**b**) Cytotoxic activity of subpopulations of NK cells on day 4 and CD4+ and CD8+ T-lymphocytes on day 6, purified from PBMC activated by M7 peptide after 20 h incubation with K562 in the presence of antibodies to GranzymeB and Fas (1:100, 20 h). (**с**) The cytotoxic activity of PBMC activated by the M7 peptide for 6 days is in the presence of a caspase 3 and 8 inhibitors, a RIP1 kinase inhibitor (necrostatin 1), and an MLKL kinase inhibitor. (*p*-value: * <0.05).

A detailed study of the cytotoxic activity of lymphocytes under the action of M7 showed that the peptide induces cytotoxicity, as was shown during incubation of full-size Mts1 with PBMC (Figure 9a). Cytotoxic activity was also blocked in the presence of anti-TREM-1 antibodies and LP17 and 17.0 inhibitors. It can be assumed that activation of lymphocytes is carried out due to the interaction of M7 with TREM-1.

A study of subpopulations of lymphocytes activated by the action of M7 showed that NK cells were predominantly activated on the 4th day of incubation of PBMC with the peptide, and CD4+ and CD8+ T lymphocytes on the 6th day. Also, as shown for full-length Mts1, NK killed HLA-negative tumor cells by secreting granzymes, inducing apoptosis in tumor cells. CD4+ and CD8+ lymphocytes used the interaction of FasL lymphocytes with the Fas receptor on tumor cells for this purpose (Figure 9b).

Activation of the Fas receptor was accompanied by the induction of apoptosis and necroptosis in tumor cells, as shown for Mts1 (Figure 9c).

Thus, the interaction of M7 with TREM-1 leads to activation of this receptor, followed by expression of inflammatory cytokine genes and activation of cytotoxic lymphocytes.

## 3. Discussion

There are two important observations in this work:

(1) A new functional activity of Mts1 in the immune response is described.
(2) A new ligand of the TREM-1 inflammatory receptor has been identified.

Mts1 is included in the S-100 group of proteins with various functional effects on cells. According to the nomenclature, its name is protein—S100A4. This protein was first discovered in metastatic tumor cells, and it has been suggested that it enhances tumor progression. Its interaction with the proteins actin and myosin, which are part of the filaments, has been shown. It is possible that these properties of Mts1 enhance the mobility of metastatic cells.

At the same time, high expression of Mts1 in cells of the immune system was shown: monocytes, NK cells, CD4+ and CD8+ T lymphocytes, and its involvement in the regulation of the immune response was established. Mts1 is present on the cell surface of CD4+ T lymphocytes and promotes the binding of the innate immunity protein Tag7 on the lymphocyte membrane to Hsp70 on the tumor cell membrane, providing cytotoxic activity of the lymphocyte. The Tag7-Mts1 complex induces the movement of cytotoxic lymphocytes to the lesion site. The cytotoxic epitope complex of Tag7-peptide 17.1 with Mts1 induces the death of TNFR1-positive tumor cells.

In this work, it was found that Mts1 binds with high specificity to the TREM-1 receptor and forms a stable complex with it. The same complex was found on the cell surface of macrophages. The appearance of soluble sTREM-1 in a conditioned medium allows us to judge the dissociation of the receptor exodomain from the cell surface during dimerization of the receptor subunits, which is considered the starting point of its activation. Based on these results, Mts1 can be considered a TREM-1 ligand.

Further activation, as shown for the Tag7, Hsp70, and HMGB1 ligands, is carried out in two directions: increased expression of proinflammatory cytokine genes and the intracellular level of these cytokines, as well as activation of cytotoxic lymphocytes that kill HLA-negative tumor cells. Interestingly, Mts1, like Tag7, Hsp70, and HMGB1, induces the same TREM-1-dependent processes in cells in the absence of an analogy in the amino acid sequence of these proteins.

One of the approaches to the detailed study of the interaction of ligands with receptors is the search for epitopes responsible for the specific binding of these multifunctional proteins to this receptor. In this work, a 17-membered peptide (ELPSFLGKRTDEAAFQK aa 41–57) was identified that is responsible for interacting with TREM-1 and inducing its biological activity. This peptide completely reproduced the activity of full-size Mts1. It interacts with TREM-1 with the same high specificity in solution and on the cell surface and induces the expression of inflammatory cytokine genes and the activation of cytotoxic lymphocytes.

It can be assumed that this peptide can be used in the creation of drugs for anti-inflammatory and antitumor therapy.

The ligands studied by us, Tag7, Hsp70, and Mts1, interact with two pro-inflammatory receptors, TREM-1 and TNFR1. The epitopes of both Tag7 and Hsp70 interacting with these receptors are located in different regions of the polypeptide chains of these ligands. However, in the case of Mts1, the M7 peptide (ELPSFLGKRTDEAAFQK aa 41–57), located in the center of the amino acid sequence, is the epitope for both receptors. To clarify this fact, it is necessary to identify the TREM-1 peptide fragment responsible for interaction with the M7 peptide and compare its structure with the structure of the TNFR1 peptide fragment interacting with this epitope.

## 4. Materials and Methods

### 4.1. Cell Cultivation and Sorting

K562, RAW264.7, U937, and Molt-4 cells were cultivated in RPMI-1640 (Himedia Laboratories Private Limited, Maharashtra, India) with 2 mM L-glutamine, 10% FCS (Invitrogen, Carlsbad, CA, USA), and 1% kanamycin (Thermo Fisher Scientific, Waltham, MA, USA). U937 was differentiated into macrophages by phorbol esters (PMA) at a concentration of 10 ng/mL, as described in [38]. L929 cells were cultured in DMEM (Himedia Laboratories Private Limited, Thane, Maharashtra, India) with 2 mM L-glutamine, 10% FCS (Cytiva Livescience™, Marlborough, MA, USA), penicillin, and streptomycin (Thermo Fisher Scientific, Waltham, MA, USA) at 37 °C in an atmosphere containing 5% CO_2_. These cell lines were obtained from the cell line collection of the N. N. Blokhin National Medical Research Center of Oncology of the Ministry of Health. Human peripheral blood mononuclear cells (PBMC) were isolated from the total leukocyte pool of healthy donors by centrifugation in a Ficoll-Paque density gradient (Cytiva Livescience™, Marlborough, MA, USA), and cultured at a density of 4 × 10^6^ cells/mL in RPMI-1640 (Himedia Laboratories Private Limited, Maharashtra, India) with Mts1 and M7 peptide (all were added in concentration of 10^−9^ M). All procedures performed were in accordance with the Declaration of Helsinki (1964) and its later amendments (World Medical Association, 2013) or comparable ethical standards and were approved by the medical ethics committee of the Institute of Gene Biology of the Russian Academy of Sciences (protocol no. 46 from 15 March 2026). CD4+ and CD8+ T-lymphocytes were isolated from the PBMC by Dynabeads™ Untouched™ Human CD4 T Cells Kit or Human CD8 T Cells Kit; NK cells were isolated from PBMC by Dynabeads™ Untouched™ Human NK Cells Kit according to the manufacturer’s protocol (Thermo Fisher Scientific, Waltham, MA, USA).

### 4.2. Proteins and Antibodies

Recombinant human Mts1 cDNA was subcloned into pQE-30 and expressed in Escherichia coli M15 (pREP4) (Qiagen, Germantown, MD, USA) and purified on a HisTrap FF (Cytiva Livescience™, Marlborough, MA, USA) 5 mL ion exchange column as described in [26]. For cellular work, Mts1 was additionally purified by HPLC gel filtration on the Superdex 75 column (Cytiva Livescience™, Marlborough, MA, USA) using the AKTA Purifier System and Unicorn 5.0 Software (GE Healthcare, Chicago, IL, USA). The resulting protein was more than 98% pure, as demonstrated by polyacrylamide gel electrophoresis. The Pierce Chromogenic Endotoxin Quant Kit (Thermo Fisher Scientific, USA) was used to test LPS quantity and detected no significant bacterial LPS contamination in the recombinant Mts1.

Recombinant sTREM-1 was obtained as described in [20].

The inhibitory peptide LP17 (LQVTDSGLYRCVIYHPP), 17.0 in concentration of 10^−8^ M, was preincubated with lymphocytes for 1 h, and then Mts1 and M7 (10^−8^ M for all) were added.

Polyclonal antibodies to Mts1 were obtained from ABclonal (ABclonal, Woburn, MA, USA), mouse monoclonal antibodies against TREM-1 were from “OOO HEMA” (“OOO HEMA”, St. Petersburg, Russia), and rabbit polyclonal antibodies to TREM-1 were obtained from Thermo Fisher Scientific (Thermo Fisher Scientific, Waltham, MA, USA).

### 4.3. Peptides

Protein Mts1 was hydrolyzed at 37 °C for 3.5 h at a 1:10 trypsin/protein ratio (*w*/*w*) in 50 mM (NH_3_)HCO_3_ (pH 8.0). The hydrolysate was then separated on a Superdex peptide column. Peptide M7 was synthesized as described in [26]. The purity of the peptides was tested by HPLC performed on the AKTA Purifier System and Unicorn 5.0 Software (GE Healthcare, Chicago, IL, USA).

### 4.4. MALDI Analysis

The MALDI analysis was performed as described in [39].

### 4.5. Cytotoxicity Assays

For cytotoxicity tests, cells of the K562 line were cultured in 96-well plates (Luoyang Fudau Biotech Co., Luoyang, Henan, China) (6 × 10^4^ per well), then lymphocytes were added (1.2 × 10^6^ per well) and incubated at 37 °C in an atmosphere with 5% CO_2_ for 3–20 h. Cytotoxicity was measured by the Cytotox 96 analysis kit (Promega, Madison, WI, USA) after 20 h of incubation in accordance with the manufacturer’s protocol. In inhibition assays, cells were pre-incubated for 1 h with the Caspase 3 inhibitor Ac-DEVD-CHO (5 μM), caspase 8 inhibitor Ac-IEID-CHO (5 μM), RIP1 (Receptor-interacting serine/threonine-protein kinase 1), kinase inhibitor necrostatin 1 (5 μM), and MLKL(Mixed lineage kinase domain like pseudokinase) kinase inhibitor (5 μM) (all from Thermo Fisher Scientific, Waltham, MA, USA).

### 4.6. Immunoassay

The level of sTREM-1 secretion was tested using the Human TREM-1 ELISA kit (Thermo Fisher Scientific, Waltham, MA, USA) in accordance with the manufacturer’s protocols. Secretion of IL-6 and TNF was measured using human ELISA Kit for Interleukin 6 (IL6) (SEA079Hu) and human ELISA Kit for Tumor Necrosis Factor Alpha (TNFα) (SEA133Hu) (both Cloud-Clone, Wuhan, China).

### 4.7. Microscale Thermophoresis

The purified sTREM-1 protein was labeled using the Alexa Fluor™ 633 Protein Labeling Kit (Life Technologies Corporation, Eugene, OR, USA) in accordance with the manufacturer’s protocol.

Mts1 and M7 peptides were analyzed using microscale thermophoresis as described in [26]. The experiments were replicated at least three times and processed using affinity analysis software (MO Control v.1.6.1, NanoTemper Technologies GmbH, München, Germany) [40].

### 4.8. RT-PCR

RNA was isolated from U937 cells after their treatment with Mts1 (10^−8^ M) and M7 peptide (10^−8^ M) for 3 h and 12 h. Each of the fractions was lysed in 500 µL of the Reagent “RNA Extract” (“Eurogen”, Moscow, Russia) according to the manufacturer’s protocol. The RNA was measured using a NanoDrop device (Thermo Fisher Scientific, Waltham, MA, USA), and then equal amounts of RNA (1.1 micrograms) were used. Electrophoresis was used to detect RNA degradation. cDNA synthesis was carried out using oligo (dT) primers (Eurogen, Russia). The products were used for cPCR with primers for genes encoding RPLP0, IL6, TNFα, and IL1β. The RPLP0 mRNA level was taken as a reference. The primers were as follows: for RPLP0: 5′-ACTGGAGACAAAGTGGGAGCC-3′ (forward) 5′-CAGACACTGGCAACATTGCG-3′ (reverse), for TNFα 5′-CTCTTCTGCCTGCTGCACTTTG-3′ (forward), 5′-ATGGGCTACAGGCTTGTCACTC-3′ (reverse); IL6 5′-AGACAGCCACTCACCTCTTCAG-3′ (forward) 5′-TTCTGCCAGTGCCTCTTTGCTG-3′ (reverse); IL1β 5′-CCACAGACCTTCCAGGAGAATG-3′ (forward) 5′-GTGCAGTTCAGTGATCGTACAGG-3′ (reverse). The measurements were carried out in at least three repetitions and the average value was calculated. Expression levels were quantified using the 2^ΔΔCt^ method.

### 4.9. Confocal Microscopy

RAW264.7 and U937 cells were grown on glass coverslips and fixed with 4% formaldehyde. Then, the cells were rinsed 3 times with PBS, and then the samples were placed into blocking solution (1% BSA in PBS) for 30 min at room temperature. Mts1 and M7 peptides were stained with polyclonal rabbit antibodies against Mts1 and Goat anti-Rabbit IgG (H + L) Cross-Adsorbed Secondary Antibody, Alexa Fluor™ 633. The TREM-1 receptor was stained with monoclonal mouse antibodies against TREM-1 and Goat anti-mouse IgG (H + L) Cross-Adsorbed Secondary Antibody, Alexa Fluor™ 488 (Molecular Probes By Life Technologies, Carlsbad, CA, USA). After washing with PBS three times at room temperature, the coverslips were embedded in ProLong Gold (Thermo Fisher Scientific, Waltham, MA, USA). Fluorescence images were obtained using a Leica STELLARIS 5 confocal microscope (Leica, Wetzlar, Germany), and then analyzed using Leica confocal software (2.61.15) and processed in ImageJ 1.54 (LOCI, Madison, WI, USA).

### 4.10. Statistical Analysis

All the results presented in the article were repeated at least three times. For significance testing, MathCad software (version 15.0, PTC, Cambridge, Great Britain) was used. Either Student criteria or a two-factor analysis of variance was used. The results are presented as average values ± SDs. The value of *p* < 0.05 was considered statistically significant. GraphPad Prism 6 software was used for data presentation.

## 5. Conclusions

Here, we have found that S100A4 (Mts1) protein is the new ligand of the known innate immunity receptor TREM-1. We have also found that a fragment of Mts1, peptide M7, also binds the TREM-1 receptor and, similar to the full-size protein, triggers the appearance of cytotoxic NK cells and T lymphocytes at different time points. Activated lymphocytes induce apoptosis and necroptosis in HLA-negative tumor cells. The new regulatory peptide may be potentially used for the regulation of inflammatory processes and activation of antitumor immunity.

## Data Availability

The original contributions presented in this study are included in the article/Supplementary Materials. Further inquiries can be directed to the corresponding author.

## Supplementary Materials

The following supporting information can be downloaded at: https://www.mdpi.com/article/10.3390/ijms27146359/s1.
