# Supplementary material for: A New Function of the S100-A4 Protein (Mts1): Mts1 Stimulates the Activation of Cytotoxic Lymphocytes via the TREM-1 Receptor
Source: Int J Mol Sci. 2026 Jul 17;27(14):6359. doi: 10.3390/ijms27146359 (PMC13409917; doi:10.3390/ijms27146359)

Supplemental materials.

Supplemental Figure S1.

Confocal micrographs of U937 cells, activated by PMA.

(a) U937 with added Mts1 protein one cell. (green -Staining with antibodies to TREM-1; red- Staining with antibodies to Mts1 and image overlay)

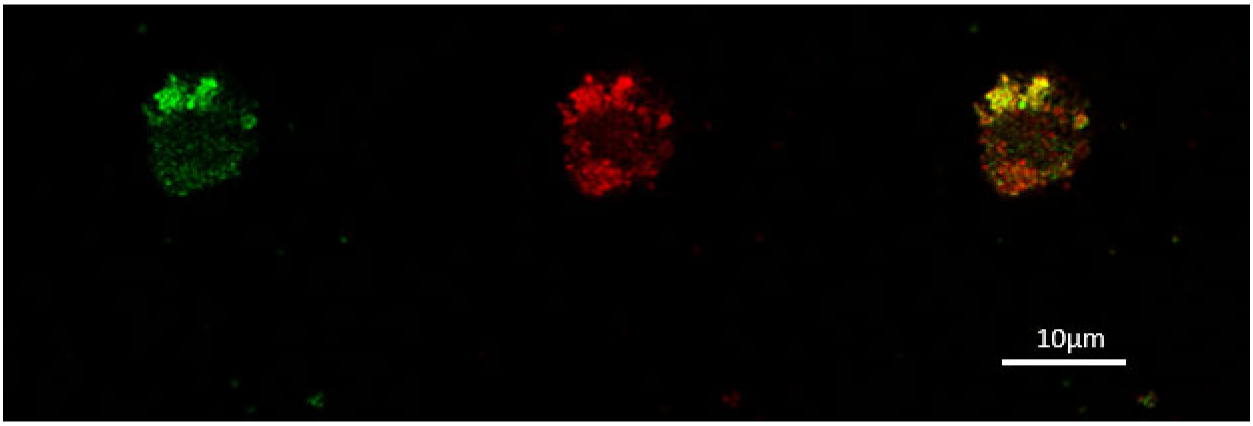

(b) U937 with added Mts1 protein –field of view. (green -Staining with antibodies to TREM-1; red- Staining with antibodies to Mts1 and image overlay)

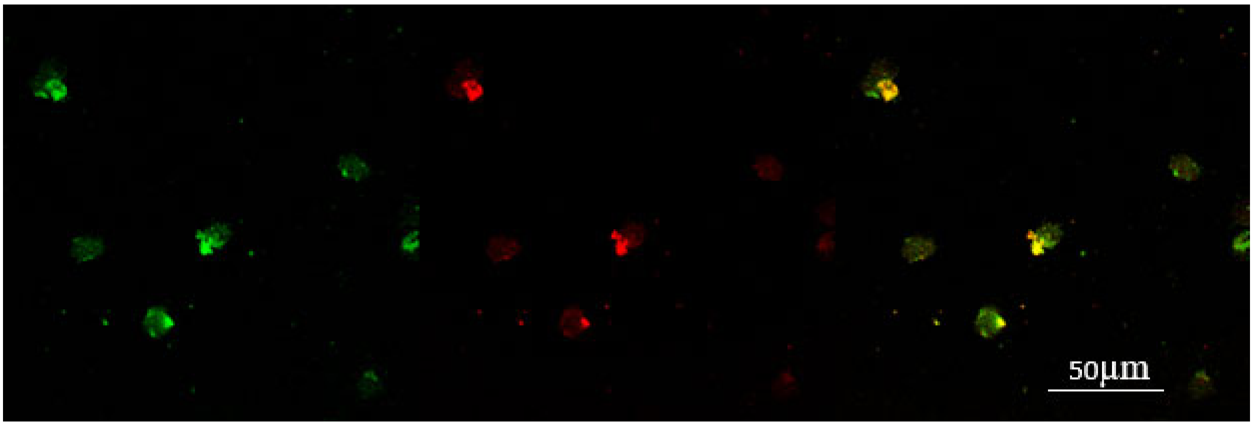

(c) U937 with added M7 peptide – one cell. (green -Staining with antibodies to TREM-1; red- Staining with antibodies to Mts1 and image overlay)

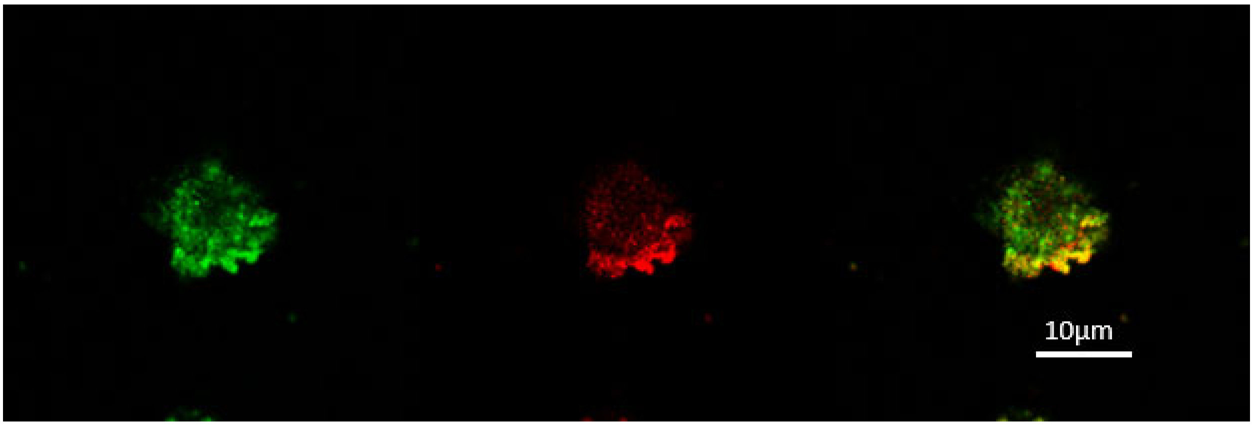

(d) U937 with added M7 protein – field of view. (green -Staining with antibodies to TREM-1; red- Staining with antibodies to Mts1 and image overlay)

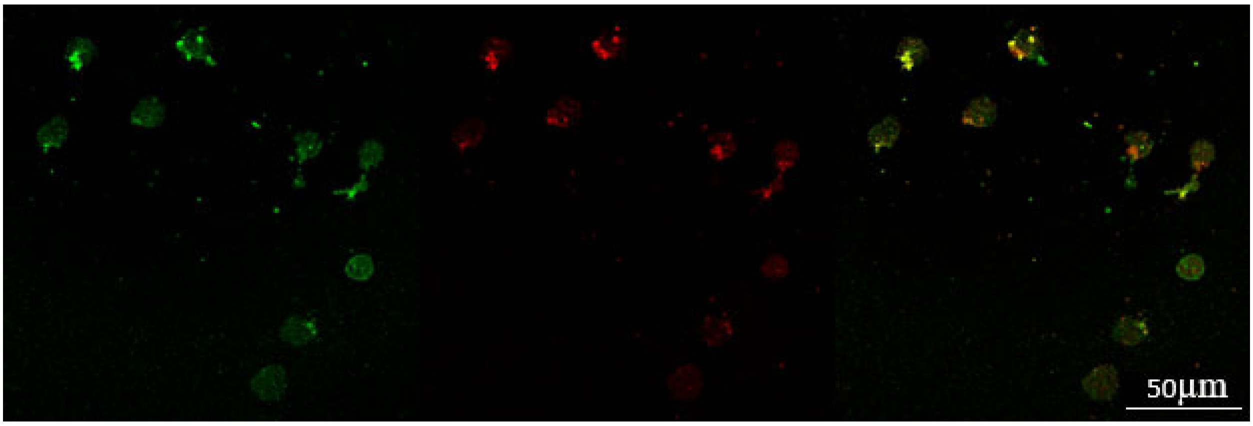

(e) Secondary antibodies control. (green -Staining with antibodies to TREM-1; red- Staining with antibodies to Mts1 and image overlay)

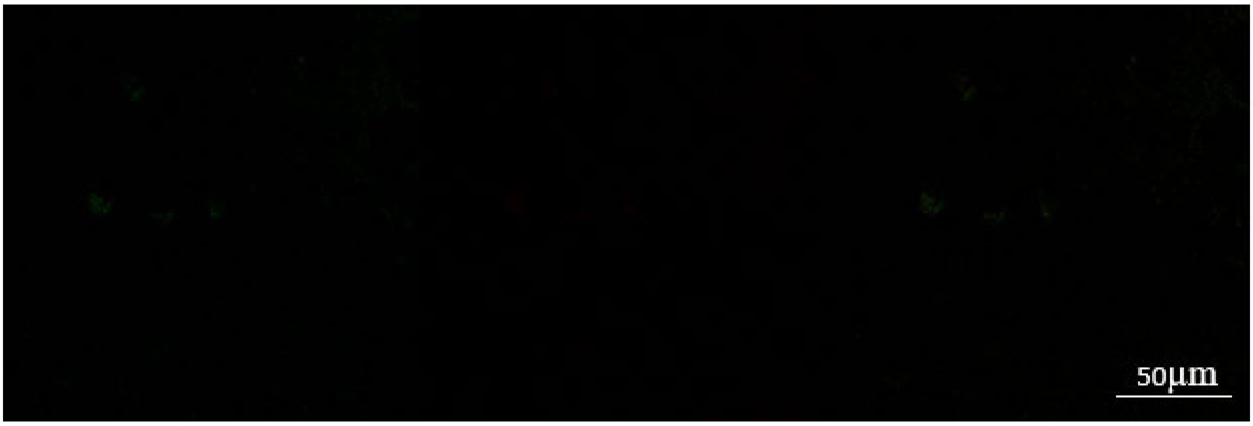

Supplemental Figure S2.

Flow cytometry data.

(a) NK cells purity on day 4 after negative magnetic separation (CD16-FITC CD56-PE)

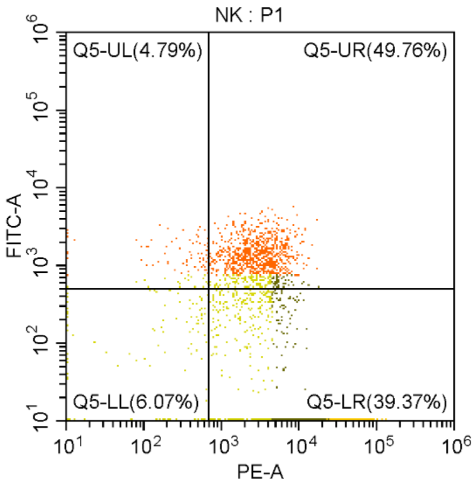

(b) CD4 T cells purity on day 6 after negative magnetic separation (CD3-FITC CD4-PE)

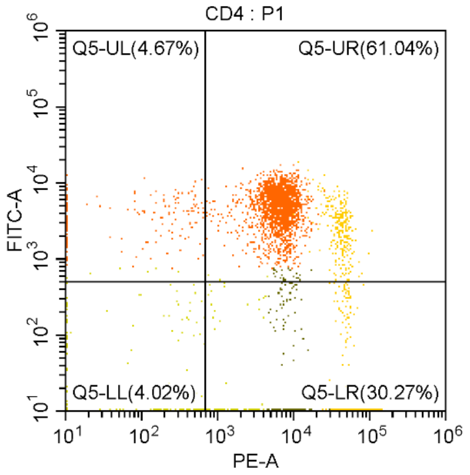

(c) CD8 T cells purity on day 6 after negative magnetic separation (CD3-FITC CD8-TC)

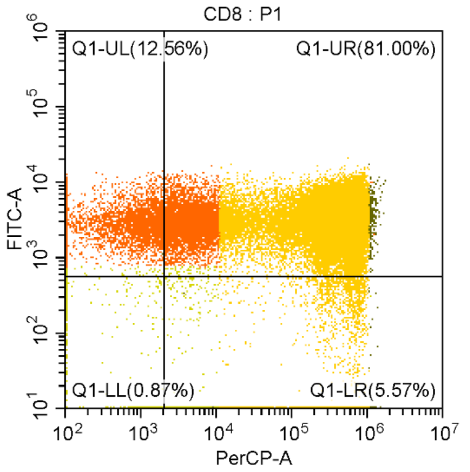

(d) FasL on CD4 T cells day 0 (left) and day 4 (right). (abFasL+antimouse FITC, CD4-PE)

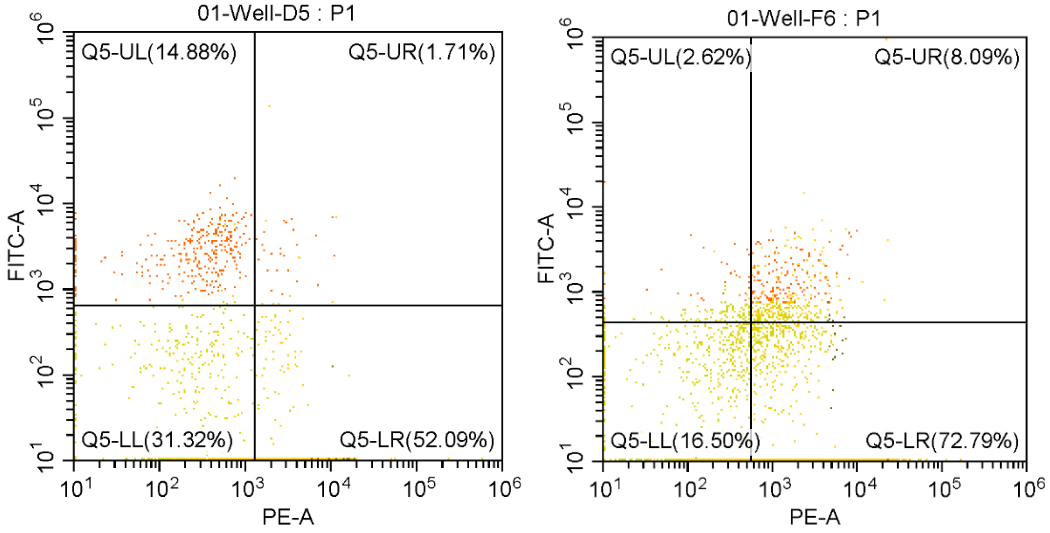

(e) FasL on CD8 T cells on day 0 (left) and day 6 (right). (abFasL+antimouse FITC, CD8-TC)

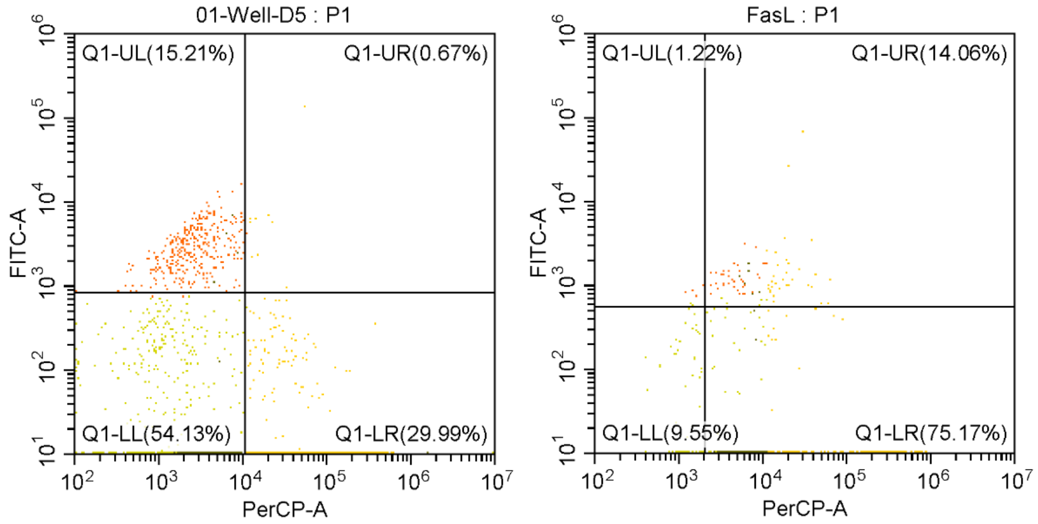

Supplemental Figure S3.

Data of pMLKL appearance in the K562 cells after their incubation with PBMC on day 6. The K562 cells were purified from cell mixture with PBMC after 1 hour of incubation with help of MicA positive magnetic separation. Purified cells were then lysed and Western blot with specific anti-pMLKL antibodies was used to identify phosphorylated form of MLKL kinase. (1 – untreated cells, 2– after incubation with Mts1 induced PBMC)

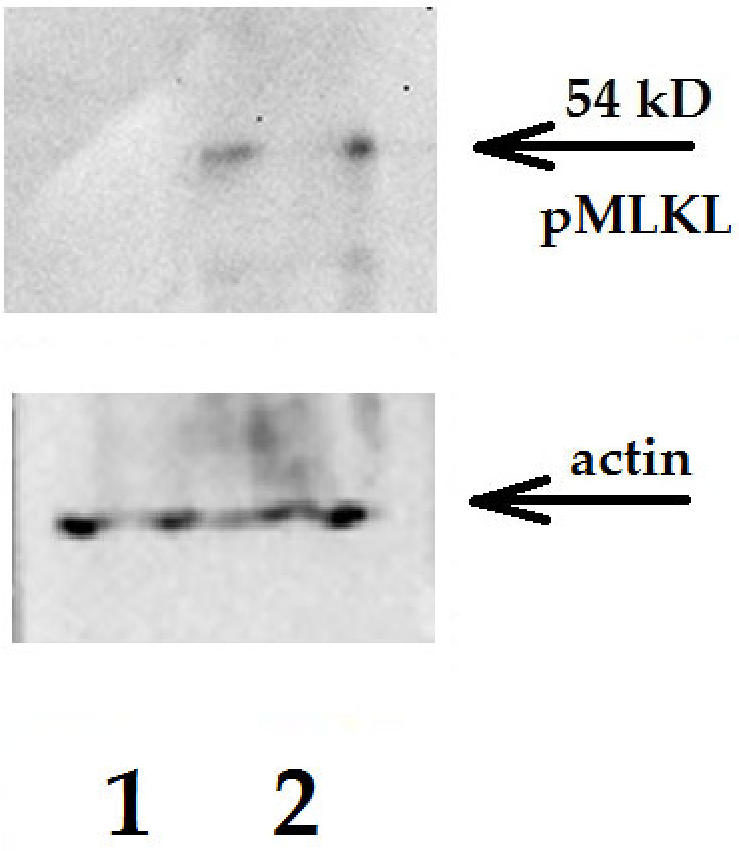

Supplement: Supplementary file 1 [file ijms-27-06359-s001.zip › ijms-4397869-supplementary.pdf]
